# Genetic polymorphisms of histone methyltransferase *SETD2* predicts prognosis and chemotherapy response in Chinese acute myeloid leukemia patients

**DOI:** 10.1186/s12967-019-1848-9

**Published:** 2019-03-28

**Authors:** Suwei Wang, Xiaoqing Yuan, Yazhen Liu, Kewei Zhu, Peng Chen, Han Yan, Daoyu Zhang, Xi Li, Hui Zeng, Xielan Zhao, Xiaoping Chen, Gan Zhou, Shan Cao

**Affiliations:** 1 Department of Clinical Pharmacology, Institute of Clinical Pharmacology, Central South University, 110 Xiangya Road, Changsha, Hunan 410008 People’s Republic of China; 2 Hunan Key Laboratory of Pharmacogenetics, Changsha, Hunan 410078 People’s Republic of China; 3 National Clinical Research Center for Geriatric Disorders, Xiangya Hospital, Central South University, Changsha, 410008 People’s Republic of China; 4 Guangdong Provincial Key Laboratory of Malignant Tumor Epigenetics and Gene Regulation, Sun Yat-Sen Memorial Hospital, Sun Yat-Sen University, Guangzhou, 510120 People’s Republic of China; 5 Department of Pharmacy, The Second Xiangya Hospital, Central South University, Changsha, Hunan 410078 People’s Republic of China; 6 Department of Hematology, Xiangya Hospital, Central South University, Changsha, Hunan 410078 People’s Republic of China; 7 National Institution of Drug Clinical Trial, Xiangya Hospital, Central South University, 110 Xiang Ya Road, Changsha, Hunan 410078 People’s Republic of China

**Keywords:** Acute myeloid leukemia, *SETD2*, Histone 3 lysine 36 trimethylation, SNP, Chemotherapy, Cytarabine

## Abstract

**Background:**

*SETD2*, the single mediator of trimethylation of histone 3 at position lysine 36, has been reported associated with initiation progression and chemotherapy resistance in acute myeloid leukemia (AML). Whether polymorphisms of *SETD2* affect prognosis and chemotherapy response of AML remains elusive.

**Methods:**

Three tag single-nucleotide polymorphisms (tagSNPs) of *SETD2* were genotyped in 579 AML patients by using Sequenom Massarray system. Association of the SNPs with complete remission (CR) rate after Ara-C based induction therapy, overall survival (OS) and relapse-free survival (RFS) were analyzed.

**Result:**

Survival analysis indicated that *SETD2* rs76208147 TT genotype was significantly associated with poor prognosis of AML (TT vs. CC + CT hazard ratio: HR = 1.838, 95% confidence interval (CI) 1.005–3.360, *p *= 0.048). After adjusting for the known prognostic factors including risk stratification, age, allo-SCT, WBC count and LDH count, rs76208147 TT genotype was still associated with OS in the multivariate analysis (TT vs. CC + CT HR = 1.923, 95% CI 1.007–3.675, *p *= 0.048). In addition, after adjusting by other clinical features, patients with rs4082155  allele G carries showed higher rate of complete remission which indicated by CR rate (AG + GG vs. AA odd ratio (OR) = 0.544, 95% CI 0.338–0.876, *p *= 0.012).

**Conclusions:**

*SETD2* genetic polymorphism is associated with AML prognosis and chemotherapy outcome, suggesting the possibility for development in AML diagnostics and therapeutics towards *SETD2*.

**Electronic supplementary material:**

The online version of this article (10.1186/s12967-019-1848-9) contains supplementary material, which is available to authorized users.

## Background

Acute myeloid leukemia (AML) is a genetically and clinically heterogeneous disorder featured by the incomplete maturation of hematopoietic stem cell and the reduction of normal blood counts [1]. Despite great efforts have been made in new therapy development, chemotherapy with cytarabine and anthracycline remains the current treatment protocols in AML, which conduct complete remission (CR) rates of 70–80% [2]. However, more than half of adult patients and around 80% of elder patients develop into primary refractoriness, relapse, or treatment-related mortality [3]. In addition, tremendous individual variability in prognosis varies greatly among patients: 5-year survival varies from 18 to 82%, and relapse rate varies from 33 to 80%, which can be partly explained by disease subtype, age, somatic mutations, gene expression abnormalities, and other molecular alterations. Genetic variants in those, such as in *NPM1, FLT3*-*ITD, CEBPA, WT1* have been verified to be marks of prognosis [4–6], however, these factors can only explain part of individual variants for AML.

Recent studies have underscored the significant role of epigenetic mechanisms in chemotherapy sensitivity and disease prognosis of AML. Such as DNA (cytosine-5)-methyltransferase 3 alpha (DNMT3A) R882 mutations, which give rise to focal hypomethylation phenotype, were associated with inferior prognosis in AML [7, 8]. Mutations in Ten–eleven-translocation (TET)-enzymes (TET2), which catalyzed the oxidation of 5-methylcytosine to 5-hydroxymethylcytosine (5hmC), confers unfavorable prognostic factor in AML patients with intermediate-risk cytogenetics [9].

*SETD2* belongs to a superfamily of lysine methyltransferase, which is a solely H3K36 trimethylation methyltransferase in mammals. H3K36 trimethylation has also been implicated in a diverse of cellular biology functions, including transcriptional activation, alternative splicing, dosage compensation, DNA replication and repair, and homologous recombination [10]. *SETD2* is a 2-hit tumor suppressor gene, for loss-of-function mutations and deletions were detected in a series of tumor types, most notably in clear cell renal cell cancer (ccRCC) [11] and high-grade gliomas [12] and subsequently presents in a subset of patients with acute lymphoblastic leukemia [13] and acute myeloid leukemia [14]. Most of mutations or abnormalities of *SETD2* has been reported to be associated with a worse outcome among patients with ccRCC suggested a protection value of *SETD2* [15]. Moreover, a selective enrichment of *SETD2* inactivating mutation in relapsed acute leukemia indicated an association with chemoresistance [16]. However, whether single-nucleotide polymorphisms (SNPs) in *SETD2* related to disease progression and drug response remains unknown.

In the present study, we performed the candidate gene association study to find out whether *SETD2* SNPs correlated with AML survival and chemotherapy response, which will explore factors leading to individual difference in AML prognosis and will provide new directions for new treatment.

## Materials and methods

### Clinical analysis sample

579 Chinese Han patients were recruited from the Department of Hematology of Xiangya Hospital from May 2009 to December 2017. All patients were diagnosed and classified based on the FAB criteria. The exclusion criteria were (1) M3 subtype, because of its specific treatment and outcomes. (2) Patients with serious diseases or other cancers, secondary leukemia and those with missing data (e.g. cytogenetics or molecular abnormalities). (3) Patients failed to follow-up were eliminated in this study. The detail therapeutic protocols were described elsewhere [17–19]. Complete remission (CR) was used to assess drug response. CR was defined according to the international recommendations, including no clinical presentation of leukemia, no evidence of extramedullary disease, bone marrow blasts < 5%, neutrophils counts > 1.0 × 10^9^/L, and platelets counts > 100 × 10^9^/L after chemotherapy [20]. Overall survival (OS) and relapse free survival (RFS) were used as disease outcomes and events were defined as any relapse or death in agreement with the criteria as previously described. All survival end points were censored at the date of last follow-up when relapse or death was not observed [21].

This study was approved by the Ethics Committee of Institute of Clinical Pharmacology of Central South University (Register No. CTXY-120025-2). Clinical study admission (Registration Number: ChiCTR-PPC-14005297) was approved by the Chinese Clinical Trial Register. Written informed consent was obtained from each patient in accordance with the recommendations of the Declaration of Helsinki and its later amendments.

### TagSNPs selection and genotyping and haplotypes

The following criteria were used to select the tagSNPs of *SETD2*. First, we chose a minor allele frequency (MAF) > 0.1 in the south Chinese population as the target according to 1000 human genomes database (https://www.ncbi.nlm.nih.gov/variation/tools/1000genomes/). Then we examined linkage disequilibrium analysis by setting r^2^ threshold at 0.8. Three tagSNPs with missense mutation were selected to represent each block (Table 1, Additional file 1: Figure S1). Polyphen database (http://genetics.bwh.harvard.edu) was used to predict potential structural and functional change of the candidate SNPs and expression quantitative trait locus (eQTL) database (https://gtexportal.org/home/) was used to predict the potential influence of the SNPs on expression the corresponding genes (Additional file 2: Figure S2).

**Table 1:** Features of three selected tag SNP from *SETD2*

SNP	Position	Alleles	Variation	MAF	Chi square	*p* ^HW^
rs4082155	3:47083895	A > G	Exon12 (Leu 1962 Pro)	0.47	0.21	0.90
rs6767907	3:47121171	G > A	Exon3 (Asn 1155 Lys)	0.35	0.44	0.80
rs76208147	3:47121396	C > T	Exon3 (Met 1080 Ile)	0.13	5.37	0.07

Genomic DNA was extracted from peripheral blood cells using E.Z.N.A.VR SQ Blood DNA Kit II (Omega Bio-Tek) according to the supplied protocol. SNP genotyping was conducted by allele-specific matrix-assisted laser desorption/ionization-time-of-flight mass spectrometry (Sequenom, San Diego, CA). About 10% of the samples were randomly selected to validate the genotypes by Sanger sequencing. The results reached to a 100% compliance. Three tagSNPs of *SETD2*, (rs4082155, rs6767907, rs76208147) were finally selected for further study.

### Statistical analysis

Statistical analysis was carried out by the software SPSS version 20.0 (SPSS INc. Chicago, IL, USA). χ^2^ test was used to determine whether genotype distribution of the SNPs were in agreement with Hardy–Weinberg equilibrium. The Kaplan–Meier curves and univariate Cox regression analysis were depicted to illustrate the profiles of OS/RFS. The independent significant factors were adjusted by multivariable analysis, the Cox proportional hazards model, including clinical factors and *SETD2* SNPs. Sensitivity analyses were conducted on CR rate. Logistic regression were used to evaluate the association between genotypes and chemotherapy response. A limited backward-selection procedure used to adjust the potential confounding covariates, including clinical factors. Statistical significance was accepted when p < 0.05 and defined two sides.

## Results

### Baseline features and overall CR status of the AML patients

The basic and clinical characteristics of the 579 AML patients in this study were recorded in Table 2. The median age of the patients was 43 (range 14–79) years old. Among these patients, 313 were male and 266 were female. The median number of WBC was 14.6 × 10^9^/L and the mean serum level of LDH was 363.5 U/L at diagnosis. Other important clinical information was also summarized in Table 2. All patients were classified into seven subtypes on the basis of FAB criteria. M3 subtype ones were ruled out because of its specific therapeutic strategy and outcome. M2 was the most common subtype (45.25%), followed by M5 (19.69%). According to cytogenetics and molecular abnormalities, 90, 262, and 112 patients were stratified as low, intermediate and high risk, respectively. A total of 368 patients (63.6%) achieved CR after one or two courses of chemotherapy, and CR could not be evaluated accurately for 33 (5.7%) patients due to insufficiencies in clinical evidence. Eighty-four (14.51%) patients received hematopoietic stem cell transplantation. All patients were followed up with a median follow-up period of 554 days (range 17–4742 days).

**Table 2:** Basic and clinical characteristics of the AML patients in this study (n = 579)

Characteristics	Value
Age at diagnosis, median (range)^a^	43 (14–79)
Gender	
Male	313 (54.1)
Female	266 (45.9)
WBC count at diagnosis (10^9^/L)^a^	14.6 (0.5–436.2)
RBC count at diagnosis (10^9^/L)^a^	2.2 (0.62–4.98)
Hemoglobin at diagnosis (g/L)^a^	72 (27–155)
Platelets count at diagnosis (10^9^/L)^a^	34 (2–1344)
Neutrophil count at diagnosis (10^9^/L)^a^	2 (0–250)
LDH at diagnosis (U/L)^a^	363.5 (17.0–9289.0)
Bone marrow blasts at diagnosis (%)^a^	71.0 (17.5–99.0)
Peripheral blood blasts at diagnosis (%)^a^	60.0 (5.0–96.0)
FBA subtype (n, %)	
M0	1 (0.17)
M1	25 (4.23)
M2	262 (45.25)
M4	98 (16.92)
M5	114 (19.69)
M6	12 (2.07)
M7	1 (0.17)
Unknown	66 (11.40)
Allo-SCT (n, %)	
Yes	84 (14.51)
No	495 (85.49)
Cytogenetics and molecular stratification	
Low risk (n, %)	90 (15.54)
Intermediate risk (n, %)	262 (45.25)
High risk (n, %)	112 (19.34)
Unknown	115 (19.86)

*Allo-SCT* allogeneic hematopoietic stem cell transplantation, *AML* acute myeloid leukemia, *FBA subtypes* French–Britain–American subtypes, *LDH* lactate dehydrogenase, *RBC* red blood cell, *WBC* white blood cell

^a^Data represents median and the range

### *SETD2* rs76208147 is associated with overall survival of AML patients

**Fig. 1:**
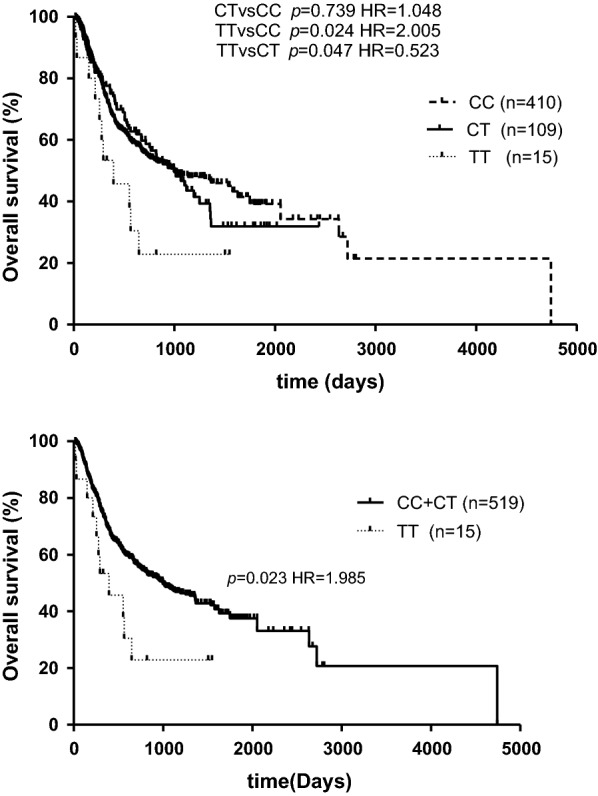
Comparison of overall survival (OS) in AML patients among genotypes of SETD2 rs76208147

Kaplan–Meier survival analysis of this series showed that *SETD2* rs76208147 rare homozygous genotype TT were associated with a worse survival compared to the common homozygous genotype CC (*p *= 0.024; Fig. 1). Because there was no significant difference of overall survival (OS) between patients with CC and CT, we combined the C allele carriers into one group to establish a recessive model. In the recessive model, rs7620147 TT genotype showed a significant poorer OS than individuals with C alleles (*p *= 0.023; Fig. 1). Univariate results showed that *SETD2* rs76208147 TT genotype was associated with a worse OS compared with the carriers of CC genotype (TT vs. CC hazard ratio: HR = 1.836, 95% confidence interval (CI) 1.001–3.369, *p *= 0.049; Table 3). As compared with patients carrying rs76208147 C allele (CC + CT), hazard ratio of OS for patients with TT genotype was 1.838 (95% CI 1.005–3.360, *p *= 0.048; Table 3). The median overall survival time of the patients with CC + CT genotypes was 885 days (95% CI 697–1073 days), which was significantly longer than those with TT genotype (Median OS = 395 days, 95% CI 71–718 days).

**Table 3:** Univariate and multivariate Cox regression analysis of SNPs associated with AML overall survival (OS)

Genotype	n	Mean ± SE (day)	Median (range, day)	HR (95% CI)	*p*	HR (95% CI)^a^	*p* ^a^
rs4082155							
AA	148	1897 ± 293	926 (663–1189)	1.00 (reference)			
AG	262	1264 ± 81	721 (478–964)	1.148 (0.867–1.520)	0.335	1.074 (0.797–1.447)	0.638
GG	120	1300 ± 114	1015 (599–1431)	1.014 (0.724–1.420)	0.989	0.989 (0.690–1.418)	0.953
rs6767907							
GG	65	1717 ± 224	817 (608–1026)	1.00 (reference)			
GA	236	1319 ± 85	735 (356–1114)	0.913 (0.714–1.167)	0.466	1.243 (0.882–1.880)	0.302
AA	229	1338 ± 162	1137 (411–1862)	1.047 (0.880–1.246)	0.602	1.261 (0.833–1.910)	0.273
rs76208147							
CC	406	1844 ± 195	817 (563–1071)	1.00 (reference)			
CT	109	1175 ± 101	1000 (697–1302)	1.001 (0.751–1.334)	0.995	1.131 (0.83–1.54)	0.428
TT	15	601 ± 146	395 (71–718)	1.836 (1.001–3.369)	0.049*	1.736 (0.84–3.58)	0.135
CC + CT	515	1806 ± 182	885 (697–1073)			1.00 (reference)	
TT	15	601 ± 146	395 (71–718)	1.838 (1.005–3.360)	0.048*	1.923 (1.007–3.675)	0.048*

*p < 0.05

^a^ Adjusted for risk stratification, age, allo-SCT, WBC count and LDH count

After adjusting for the known prognostic factors including risk stratification, age, allo-SCT, WBC count and LDH count (adjusted p < 0.001, Additional file 3: Table S1), rs76208147 TT genotype was still associated with worse OS in the multivariate analysis (TT vs. CC + CT HR = 1.923, 95% CI 1.007–3.675, *p *= 0.048, Table 3).

However, other tagSNPs, rs4082155 and rs6767907 did not associated with OS of AML patients in univariate analysis (Table 3). When RFS was used for endpoint, no significantly tagSNPs of *SETD2* were observed through univariate and multivariate analysis.

### Candidate SNPs are associated with AML Ara-C based chemotherapy

The CT genotype of rs76208147 showed lower chemotherapy resistant risk compare with CC genotype (OR = 0.567, 95% CI 0.35–0.919, *p *= 0.021, Table 4). The CR rate were 64.7% and 76.4% for CC and CT genotypes, respectively. This SNP did not passed the correction by adjusted for age, risk stratification, LDH and WBC (adjusted p < 0.05, Additional file 4: Table S2) in logistic regression analysis. However, when rs76208147 TT genotype was taken into account, the *p* value failed to meet the statistically difference (OR = 0.500, 95% CI 0.14–1.82, *p *= 0.293). This may result from the small sample size, for only three patients involved in the non-CR group with TT genotype. To be noted, homozygous carriers of the allele T has the same tendency with CT genotype indicated by the percentage of CR patients in TT genotype is much higher than CC genotype.

**Table 4:** Comparison of CR rate among genotypes after two courses of Ara-C based chemotherapy

Genotype	Total (n)	CR (n, %)	Non-CR (n, %)	OR (95% CI)	p	OR (95% CI)^a^	p^a^
rs4082155							
AA	157	96 (61.1%)	61 (38.9%)	1.00 (reference)		1.00 (reference)	
AG	266	184 (69.2%)	82 (30.8%)	0.701 (0.464–1.060)	0.092	0.562 (0.340–0.931)	0.025*
GG	123	88 (71.5%)	35 (28.5%)	0.626 (0.377–1.039)	0.07	0.501 (0.265–0.946)	0.033*
AA	157	96 (61.1%)	61 (38.9%)	1.00 (reference)		1.00 (reference)	
AG + GG	389	272 (69.9%)	117 (30.1%)	0.677 (0.460–0.997)	0.048*	0.544 (0.338–0.876)	0.012*
rs6767907							
GG	237	155 (65.4%)	82 (34.6%)	1.00 (reference)		1.00 (reference)	
GA	242	171 (70.7%)	71 (29.3%)	0.698 (0.396–1.230)	0.213	0.668 (0.323–1.379)	0.275
AA	67	42 (62.7%)	25 (37.3%)	0.889 (0.506–1.560)	0.681	1.126 (0.548–2.317)	0.746
rs76208147							
CC	422	273 (64.7%)	149 (35.3%)	1.00 (reference)		1.00 (reference)	
CT	110	84 (76.4%)	26 (23.6%)	0.567 (0.350–0.919)	0.021*	0.600 (0.342–1.053)	0.075
TT	14	11 (78.6%)	3 (21.2%)	0.500 (0.137–1.819)	0.293	0.545 (0.114–2.604)	0.447

*p < 0.05

^a^ Adjusted for age, risk stratification, LDH and WBC

For the *SETD2* rs4082155, allele G carriers showed significant higher sensitivity of chemotherapy than AA carriers (GG + AG vs. AA: OR = 0.677, 95% CI 0.460–0.997, *p *= 0.048) in the dominant model. Logistic regression analysis showed significant associations of risk stratification, age, pretreatment WBC counts and LDH levels with non-CR risk (Additional file 4: Table S2). When adjusted by these risk factors, notably, rs4082155 allele G carriers was still significantly affected chemotherapy sensitivity. Compared with AA genotype, both AG and GG genotype exhibited lower risk of chemoresistance (AG vs. AA: OR = 0.562, 95% CI 0.340–0.931, *p *= 0.025; GG vs. AA: OR = 0.501, 95% CI 0.265–0.946, *p *= 0.033, Table 4). After combining AG and GG genotypes into one group to create a dominant model, rs4082155 allele G carriers indicated more significant higher chemotherapy sensitivity than AA genotype (AG + GG vs. AA: OR = 0.544, 95% CI 0.338–0.876, *p *= 0.012). In the carriers of three genotypes of rs4082155, the ratio of complete remission was 61.1% (AA), 69.2% (AG), and 71.5% (GG) respectively.

### Haplotype analysis

We further studied the haplotype of these three SNPs of *SETD2* using PHASE 2.0 software and analyzed its association with clinical outcome and chemotherapy. Three haplotypes with frequencies higher than 5% were selected. The frequencies of haplotypes AGC (haplotype 1), GAC (haplotype 2), GGT (haplotype 3) were 53.7%, 34.4%, 11.9%, respectively. Haplotype GGT with one or two copy number presents better chemotherapy response compared with no copy number (OR: 0.576, 95% CI 0.38–0.88, *p *= 0.01, Table 5). No significant association was observed between chemotherapy and other haplotypes. The association between these three haplotypes and OS/RFS was analyzed by log-rank test in univariate analysis. However, no haplotype can be a predictable factor for OS or RFS of patients.

**Table 5:** Haplotype analysis associated with chemosensitivity in *SETD2* polymorphism

Haplotype	CR (n, %)	NR (n, %)	χ^2^	*p*	OR (95% CI)
AGC	188 (50.9%)	102 (56.6%)	2.871	0.090	0.803 (0.623–1.035)
GAC	127 (34.3%)	62 (34.5%)	0.000	0.998	1.000 (0.767–1.303)
GGT	51 (13.9%)	16 (8.4%)	6.679	0.010	1.738 (1.138–2.652)

## Discussion

In this study, we studied the association between *SETD2* tagSNPs with chemosensitivity response to Ara-C baseds therapy as well as disease prognosis in Chinese AML patients for the first time. We found that *SETD2* rs76208147 TT genotype predicted worse OS in the AML patients, while the *SETD2* rs4082155 AA genotype were associated with chemoresistance after Ara-C based therapy. Moreover, haplotype with one or two copies of GGT showed better chemotherapy response compare with individuals with no copies.

The *SETD2* gene encodes a 230 kDa protein that is non-redundantly responsible for trimethylation of lysine 36 on histone H3 (H3K36me3), a critical mark that is involved in various important cellular processes such as transcriptional elongation, alteration splicing, mismatch repair regulation and homologous recombination repair [22–27]. Recently, *SETD2* was identified as tumor suppressor, as loss-of-function mutation with *SETD2* has been discovered in various tumors, including ccRCC, lung adenocarcinoma, gliomas, AML, ALL, and mastocytosis [12, 28–32]. In addition, loss-of-function mutation in *SETD2* and/or decreased H3K36me3 levels have been linked to poor clinical prognosis in lung cancer and ccRCC [15, 33]. In AML, *SETD2* mutations are recurrent events and are associated with chromosomal abnormalities that are known to be driver mutations in leukemogenesis, such as MLL-rearrangement [14]. In the presence of chromosomal translocation, such as MLL-rearrangement, knockdown of *SETD2* promotes initiation as well as progression of tumor by expediting the potential of self-renewal of leukemic stem cell [14]. Notably, under normal hematopoietic condition, *SETD2* is required to maintain self-renewal capability of hematopoietic stem cell, *SETD2*-deleted HSCs gives rise to malignant transformation eventually [34]. Consistent with our findings, *SETD2* rs76208147 TT genotype indicates worse prognosis of AML patient, which underlying mechanism warrants further investigation.

Recently, it is well accepted that *SETD2* was associated with chemotherapy sensitivity. Studies have identified the enrichment of mutations in *SETD2* in relapsed acute lymphoblastic leukemia and MLL-rearranged acute leukemia [16]. In addition, *SETD2* mutations led to resistance to DNA-damaging agents, cytarabine, 6-thioguanine, doxorubicin, and etoposide, but not to a non-DNA damaging agent via impairing DNA damage recognition [35]. Moreover, acquired loss-of-function mutations in *SETD2* enable metastatic non-small cell lung cancer to resist to cisplatin [36]. All of these reports suggested that *SETD2* exerted a subtle impact on the DNA mismatch repair (MMR) machinery [37]. It has been reported that MSH6, which is an essential component of the MMR machinery localizes to chromatin by binding to the H3K36 trimethyl mark that *SETD2* makes. *SETD2* knockdown has been shown to give rise to mislocalization of MSH6 and microsatellite instability and a mutator phenotype in several cell types. There are at least two possible explanations to link *SETD2* inactivation to the survival of leukemia cell. First, a mutator phenotype induced by *SETD2* inactivation could increase the mutational diversity and thus, adaptability of the leukemia, leading to clonal survival. Two, since intact MMR is important for triggering apoptosis and/or cell cycle arrest in response to many DNA damaging chemotherapies, *SETD2* loss may lead to chemotherapy tolerance [35]. To be mentioned, the function of *SETD2* is also involved in homologous recombination which deficiency have generally led to sensitivity to DNA-damaging agents, such as cisplatin [24]. That being said, the impact of *SETD2* alteration on homologous recombination require further investigation.

*SETD2* was discovered because of its capability to catalyze H3K36 trimethyltransferase, and major researches have been confined to its role of histone modification. But, in recent studies it has become apparent that *SETD2* exerts diverse functions that unrelated to histone modification. For instance, it has been reported that *SETD2* directly mediates STAT1 methylation on lysine 525, which amplifies the antiviral immunity of IFN-a [38], and a-tubulin methylation on lysine 40, which maintains genomic stability through microtubule methylation [39]. Taken together, the contributions of these additional functions of *SETD2* on prognosis and treatment of AML remain to be fully elucidated.

There are several limitations in the present study. First, the outcomes of our study failed to undergo multiple test adjusting, P values lost statistical significance when Bonferroni correction was performed possibly due to the limited sample size included in our study. Second, Ara-C response is affected by various genetic factors, the contribution of single unique gene to the drug exposure might be limited.

## Conclusion

In conclusion, our study showed for the first time that SNPs in epigenetics modulator genes such as *SETD2* are associated with drug sensitivity to Ara-C based chemotherapy and prognosis in Chinese AML patients. As the factors applicable for prediction of AML outcome are still limited, our findings provide insightful information that SNPs in epigenetic modulator may be considered as potential markers in evaluation of AML outcome. For *SETD2* involving in tumor initiation, progression as well as chemosensitivity through different mechanism. Functional studies are now warranted to illustrate the exact biological function of *SETD2* in AML, but our data add to a growing body of evidence suggesting a role for *SETD2* and H3K36me3 in AML that may be developed for the exploitation of novel therapeutic target.

## Additional files

**Additional file 1: Figure S1.** Position of three SNPs in SETD2.

**Additional file 2: Figure S2.** eQTL analysis of SETD2 polymorphisms.

**Additional file 3: Table S1.** Multivariate Cox regression analysis of clinical characteristics impacting AML OS.

**Additional file 4: Table S2.** Unconditional logistic regression analysis of clinical features related to non-CR risk in AML.
