# Supplementary figures and images for: Genetic polymorphisms of histone methyltransferase SETD2 predicts prognosis and chemotherapy response in Chinese acute myeloid leukemia patients
Source: J Transl Med. 2019 Mar 28;17:101. doi: 10.1186/s12967-019-1848-9 (PMC6437967; doi:10.1186/s12967-019-1848-9)

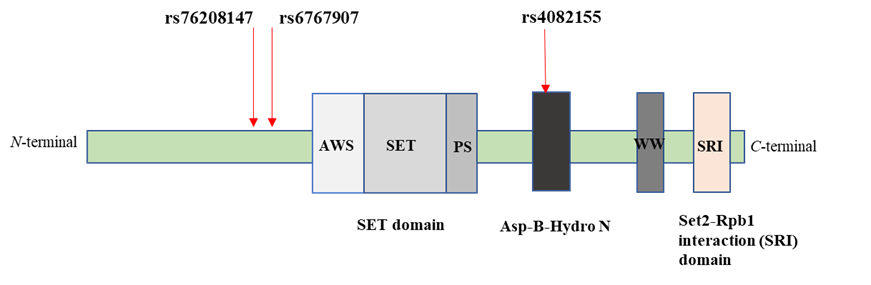


Additional file 1: Fig. S1 Position of three SNPs in SETD2.

Supplement: Supplementary file 1 — Additional file 1: Figure S1. Position of three SNPs in SETD2. [file 12967_2019_1848_MOESM1_ESM.docx]

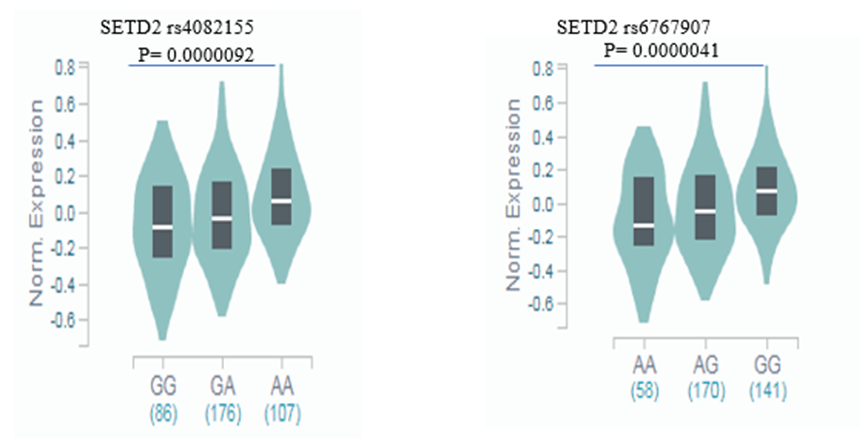


Additional file 2: Fig S2. eQTL analysis of SETD2 polymorphisms.

Supplement: Supplementary file 2 — Additional file 2: Figure S2. eQTL analysis of SETD2 polymorphisms. [file 12967_2019_1848_MOESM2_ESM.docx]
