# Supplementary material for: Genetic polymorphisms of histone methyltransferase SETD2 predicts prognosis and chemotherapy response in Chinese acute myeloid leukemia patients
Source: J Transl Med. 2019 Mar 28;17:101. doi: 10.1186/s12967-019-1848-9 (PMC6437967; doi:10.1186/s12967-019-1848-9)
Supplement: Supplementary file 3 — Additional file 3: Table S1. Multivariate Cox regression analysis of clinical characteristics impacting AML OS. [file 12967_2019_1848_MOESM3_ESM.docx]

**Additional file 3: Table S1.** Multivariate Cox regression analysis of clinical characteristics impacting AML OS

| Variables in the model | HR (95% CI) | *P* |
| --- | --- | --- |
| Gender | 1.131(0.87-1.47) | 0.359 |
| Age | 1.012(1.00-1.02) | 0.019 |
| Allo-SCT | 0.073(0.03-0.18) | 9.9385E-9 |
| LDH | 1.000(1.00-1.00) | 3.12E-4 |
| WBC | 1.002(1.00-1.00) | 0.030 |
| Risk stratification groups |  |  |
| Intermediate vs High | 0.254(0.11-0.46) | 3.89E-4 |
| Low vs Intermediate | 0.429(0.28-0.65) | 8.5E-5 |
