# Supplementary material for: Genetic polymorphisms of histone methyltransferase SETD2 predicts prognosis and chemotherapy response in Chinese acute myeloid leukemia patients
Source: J Transl Med. 2019 Mar 28;17:101. doi: 10.1186/s12967-019-1848-9 (PMC6437967; doi:10.1186/s12967-019-1848-9)
Supplement: Supplementary file 4 — Additional file 4: Table S2. Unconditional logistic regression analysis of clinical features related to non-CR risk in AML. [file 12967_2019_1848_MOESM4_ESM.docx]

**Additional file 4: Table S2.** Unconditional logistic regression analysis of clinical features related to non-CR risk in AML

| Variables in the model | HR (95% CI) | *P* |
| --- | --- | --- |
| Age | 0.985(0.96-0.99) | 0.025 |
| LDH | 1.000(0.99-1.00) | 0.015 |
| WBC | 0.987(0.98-1.00) | 0.005 |
| Risk stratification groups |  |  |
| Intermediate vs High | 0.254(0.11-0.45) | 0.121 |
| Low vs Intermediate | 0.443(0.25-0.97) | 0.043 |
